# Circular to the Medical Profession of Tompkins, Tioga, and Broome Counties, N. Y.

**Published:** 1847-09

**Authors:** 


*Circular to the Medical Profession of Tompkins, Tioga, and Broome Counties, N. Y.* We publish, with pleasure, the following Circular, and hope that the Convention will be well attended :—

CIRCULAR:

DEAR SIR,—At the semi-annual meeting of the Cortland County Medical Society, held on the 16th ult., the following preamble and resolution were unanimously adopted :

"Whereas it has become necessary that every intelligent and honest member of the Medical Profession in this country should exert his utmost influence not only to sustain, but to elevate his profession ; and inasmuch as these objects can be most efficiently secured, by the associate influence of a common brotherhood, therefore,

*Resolved*—That the Cortland County Medical Society appoint a committee to prepare and send a circular to the individual members of the profession in the counties of Tompkins, Tioga, and Broome, inviting their co-operation in the organization of a District Medical Association."

The undersigned were appointed a committee to carry out the wish of the Society as contemplated in the above resolution. The committee would add, however, that this proceeding of our Society was not entered into with the intention of officiously taking the lead in the formation of the organization proposed, but that it was in accordance with the unanimous recommendation of the New York State Medical Society, at its annual meeting in February last, as expressed in the following resolution :

"*Resolved*—That as far as it may be deemed practicable, it be recommended that voluntary associations of Physicians be formed, whose duty it shall be to hold frequent meetings, to read dissertations on medical subjects ; to relate interesting cases of disease ; and interchange views with each other on all subjects connected with our profession ; that thus, interesting and instructive matter may be in readiness to be laid before the respective County Societies at their regular meeting, which will tend to call out the latent talent of the profession, and make our meetings something more than mere medical elections, while at the same time it will make manifest before the public, the wide difference between science and quackery."

The Committee would also take the liberty of calling your careful attention to the National Medical Association recently formed in Philadelphia, and to its plans for the sustension and elevation of the Medical Profession of the United States ; believing, that an attentive consideration of the views projected by the National Medical Convention in relation to the elevation of our ethical and educational standard in this country would inspire you with still greater confidence in the truly noble protesssion of your choice, and encourage you to pursue with still greater ardor the cultivation of medicine and the collateral sciences.

But the duty of the Committee is mainly to call your attention to the propriety of forming a "District Medical Association," to embrace the counties of Tompkins, Tioga, Broome and Cortland. In relation to this subject we invite your free correspondence. The Committee would respectfully suggest, however, that in the event of a general approval of the formation of such an Association by the members of the profession in the above named counties, the Convention for its formation be held in Owego, (on account of its centrality and accessibility,) and that the time of meeting be some day in September next. On this point, also, we hope you will be free to extend us your advice.

F. HYDE,

G. W. BRADFORD, } *Committee.*

C. GREEN.

HOMER, July 8, 1847.

P. S.—Since the above went to press, the Committee have had the pleasure of being officially informed that the Tioga County Medical Society has passed a resolution similar to the one above noticed, recommending the meeting of the Convention at Owego, on the second Tuesday of September next. The Committee would, therefore, take the liberty, without further correspondence, of appointing the meeting of the Convention at Owego, on Tuesday, the 14th day of September next, at 1 o'clock in the afternoon.

